# Gameplay as a Source of Intrinsic Motivation in a Randomized Controlled Trial of Auditory Training for Tinnitus

**DOI:** 10.1371/journal.pone.0107430

**Published:** 2014-09-12

**Authors:** Derek J. Hoare, Nicolas Van Labeke, Abby McCormack, Magdalena Sereda, Sandra Smith, Hala Al Taher, Victoria L. Kowalkowski, Mike Sharples, Deborah A. Hall

**Affiliations:** 1 National Institute for Health Research Nottingham Hearing Biomedical Research Unit, Nottingham, United Kingdom; 2 Otology and Hearing group, Division of Clinical Neuroscience, School of Medicine, University of Nottingham, Nottingham, United Kingdom; 3 Institute of Educational Technology, The Open University, Milton Keynes, United Kingdom; 4 Medical Research Council Institute of Hearing Research, Nottingham, United Kingdom; University of Regensburg, Germany

## Abstract

**Background:**

Previous studies of frequency discrimination training (FDT) for tinnitus used repetitive task-based training programmes relying on extrinsic factors to motivate participation. Studies reported limited improvement in tinnitus symptoms.

**Purpose:**

To evaluate FDT exploiting intrinsic motivations by integrating training with computer-gameplay.

**Methods:**

Sixty participants were randomly assigned to train on either a conventional task-based training, or one of two interactive game-based training platforms over six weeks. Outcomes included assessment of motivation, tinnitus handicap, and performance on tests of attention.

**Results:**

Participants reported greater intrinsic motivation to train on the interactive game-based platforms, yet compliance of all three groups was similar (∼70%) and changes in self-reported tinnitus severity were not significant. There was no difference between groups in terms of change in tinnitus severity or performance on measures of attention.

**Conclusion:**

FDT can be integrated within an intrinsically motivating game. Whilst this may improve participant experience, in this instance it did not translate to additional compliance or therapeutic benefit.

**Trial Registration:**

ClinicalTrials.gov NCT02095262

## Introduction

Tinnitus refers to a person's perception of sound in the ears or head despite any corresponding sound in the external world. Affecting 10–15% of the population [1] it represents a major healthcare burden [2] yet its underlying mechanisms are not well understood, and there is no uniformly effective treatment. Hearing loss is typically comorbid with tinnitus suggesting the tinnitus percept is a direct consequence of maladaptive neuroplastic responses to hearing loss [3]. Current models of tinnitus generation therefore focus on the potential consequences of hearing loss on neuronal activity within the central auditory system [4]–[12], although neuronal structures or networks responsible for tinnitus that are independent of those for hearing loss are yet to be convincingly determined [13]–[16].

The different models of tinnitus generation provide various potential regimes for novel tinnitus intervention. One current mainstay in tinnitus management is the provision of passive sound stimulation to mask the tinnitus sound [17]–[18]. However, active forms of sound enrichment such as Frequency Discrimination Training (FDT) are more recently proposed as interventions to *interrupt* tinnitus generation and maintenance in a targeted way, rather than just mask it [19]. Early studies of FDT for tinnitus have all based their hypotheses on a cortical reorganization model of tinnitus typically citing the seminal work of Recanzone et al. [20], where perceptual learning through *active* listening appeared key to functional reorganisation of the auditory cortex [21]–[23]. A more recent animal study from Engineer et al. [10], again suggested that passive sound exposure alone was not sufficient. They reported neuroplastic and behavioural changes associated with noise-induced hearing loss were reversed through passive sound stimuli when paired with electrical stimulation of the vagus nerve. Whilst sound stimulates the auditory cortex, vagal nerve stimulation is claimed to promote neuromodulator release akin to that generated by the use of behavioural reward to reinforce behavioural or neurophysiological change due to learning [24]–[25].

We recently reviewed studies of auditory training for tinnitus concluding the need for good quality trials to reliably estimate its potential as a therapy [19] and have since published our own investigation of the effects of FDT in adults with tinnitus [26]. In this study we compared training at hearing loss frequencies to training at normal hearing frequencies. Whilst overall we saw a clinically significant improvement in tinnitus handicap after training, this effect was independent of whether training stimuli were in the region of hearing or hearing loss. We therefore concluded that training at *any* frequencies could equally result in some generic improvement in tinnitus self-report and hypothesised the improvement to be cognitive, by reducing deficits in attention for example, rather than physiological.

Discrepant between animal studies and human studies of FDT for tinnitus however is the use of reward. Human studies of FDT for tinnitus have thus far derived training from *n*-alternative forced-choice paradigms conventionally used to determine psychophysical threshold for discrimination [27]–[28]. As such, reinforcement of experience with reward was not considered. Participants were simply required to ‘react’ to the stimulus presentation rather than ‘interact’ with the training programme. Participants presumably took part because of the potential for improvement in their tinnitus (extrinsic motivation). It may be that more interactive forms of FDT that incorporate elements of gameplay such as decision making, strategy development, competition, which are intrinsically (top-down) motivating would make training more rewarding and yield significant further benefit for patients [29]. In the health domain, computer-assisted educational health interventions are shown to be more effective when they support basic patient needs such as the desire for greater autonomy [30]. Furthermore it is proposed that future intervention-focused studies evaluating the influence of video games on health should account for the need for satisfaction provided by these games [31]. In the case of FDT for tinnitus, where the ‘material’ used is typically short pips of pure tones, we perhaps need to meet baser needs such as measurable enjoyment and engagement with the game for maximum therapeutic benefit to be realised. For the current study therefore we developed two training platforms where the core listening task involved FDT but we systematically introduced gaming elements. These elements were expected to provide intrinsic motivation through challenge (use of point scoring, target scores), control (opportunity for developing personal game strategy), fantasy (integration of the training task and the perceived objective of the game), and curiosity, that would motivate and reward participants [32].

We hypothesised that interactive games would prove more intrinsically motivating, and lead to greater improvement in self-reported tinnitus handicap than in previous studies, and to improvement in cognitive performance. Two main questions are addressed in this study; (1) can we use gameplay to make FDT intrinsically motivating, and (2) does FDT delivered in a gaming format have significant therapeutic benefit over training delivered in a reactive task-only format?

## Methods

This work is reported according to the CONSORT statement for randomized trials of non-pharmacological treatments [33] (Checklist S1). The work was initially conceived as an experimental study but to comply with requirements for publication the study was registered as a clinical trial after enrolment of participants started (Clinicaltrials.gov ID: NCT02095262) (Protocol S1). The authors confirm that there are no ongoing or related trials for this intervention.

All testing took place at the NIHR Nottingham Hearing Biomedical Research Unit.

### Ethics statement

Ethical approval for this study was granted by the National Research Ethics Service for England (Nottingham Research Ethics Committee). Participants gave their written informed consent to take part in the study in accordance with the approval granted.

### Participants

Sixty participants were recruited through advertisement in local Ear, Nose & Throat and audiology departments, and on our departmental website. Participants were adults with chronic subjective tinnitus who had a ≥20 dB hearing loss on at least one test frequency (0.125, 0.5, 1, 1.5, 2, 3, 4, 6, 8, 9, 10, 11.25, 12.5, 14 kHz) in at least one ear, and were not currently receiving any therapy or other intervention that could impact their hearing or tinnitus. Participants with hearing loss ≥40 dB at all test frequencies were excluded as not being able to sufficiently hear the training stimuli. The screening assessment included a case history, the Beck Anxiety Index and Beck Depression Index [34] and the Hyperacusis Questionnaire [35]. Participants with clinically significant scores on any of these questionnaires were excluded as requiring clinical intervention.

### Sample size

In our previous study [26] it was estimated that 14 participants per group would be required to detect a statistical difference in mean THQ score change between two groups. Baseline tinnitus handicap was different between groups in our previous study however, suggesting that a larger sample size would be more appropriate. We therefore aimed to recruit 20 participants to each group which according to Cohen [36]–[37] would generally be required for significance in a one-sided test at alpha 0.05 and a power of 0.8. A one-sided test was considered appropriate as we had a directional prediction of benefit for all three groups.

### Allocation of Participants to Training Groups

**Figure 1:**
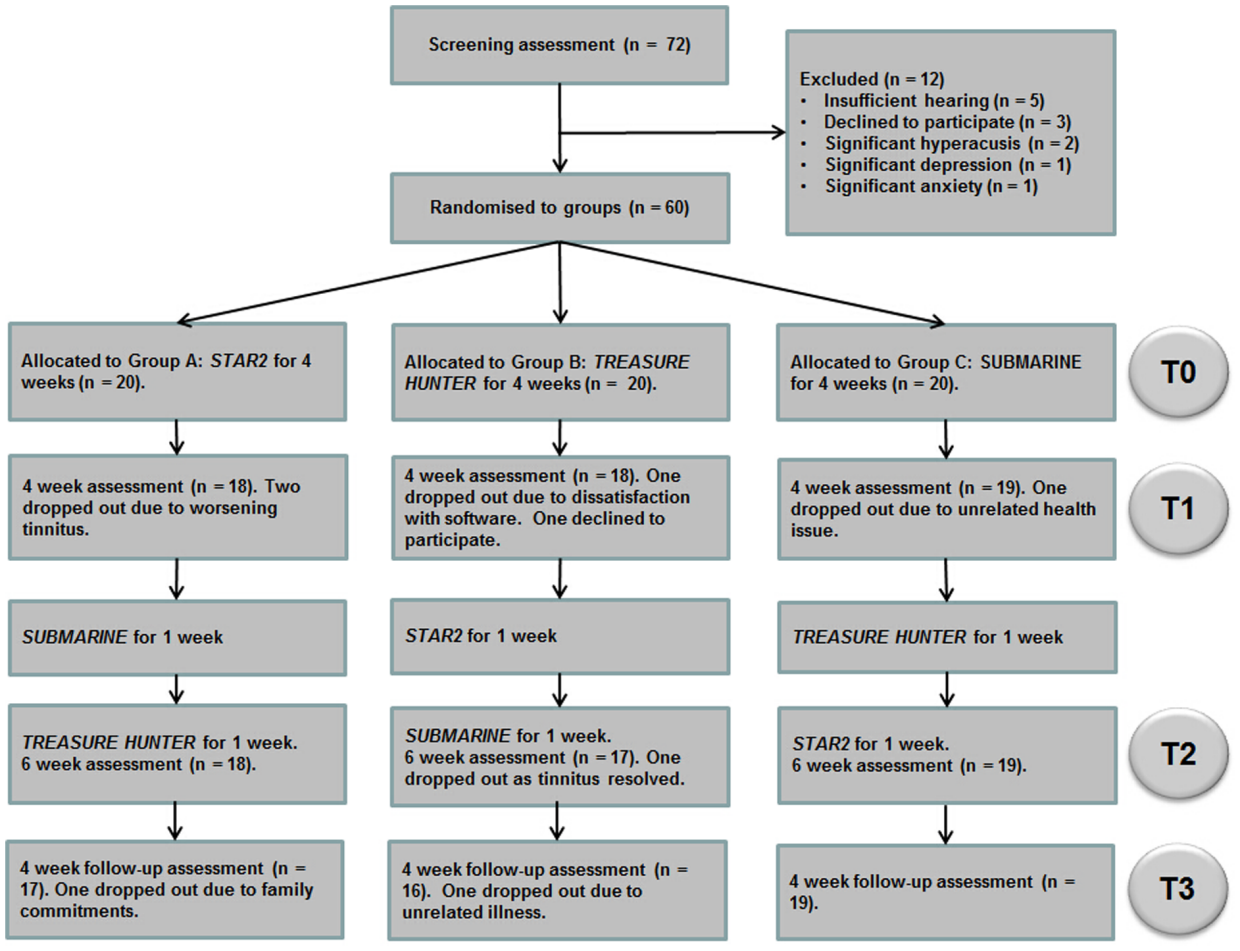
Trial flow chart.

Participants were randomized using a minimization protocol [38] to ensure groups were balanced with respect to (i) severity of tinnitus; THQ score; <600, 600–1200, >1200, (ii) age; 18–49, 50–69, 70+, and (iii) gender. A number of steps ensured blinding of the outcome assessment. First, the minimization was performed by an independent researcher who was not otherwise involved in the study. Second, assessment of tinnitus was carried out by a researcher who did not know which group the participant was allocated to. Third, the researcher who programmed the laptops and instructed the participants was kept unaware of any changes in the participant's tinnitus throughout the study. Participants were not blinded but received the same generic information about the purpose of the study and were required not to discuss their tinnitus or gameplay experience where it would compromise study blinding of outcome assessment. Figure 1 shows the flow of participants through the study, exclusions, and dropout.

### Audiometry

Pure-tone audiometry was conducted in a sound-proofed booth using the Siemens Unity 2 system and Sennheiser HAD 200 headphones. The frequency range tested was 0.125 kHz to 14 kHz. Pure tone average was calculated as the average threshold across 0.5, 1, 2, and 4 kHz for both ears. Audiometry was conducted at the initial assessment and again at visit three to check the stability of hearing thresholds.

### Frequency discrimination training (FDT) regimes

**Figure 2:**
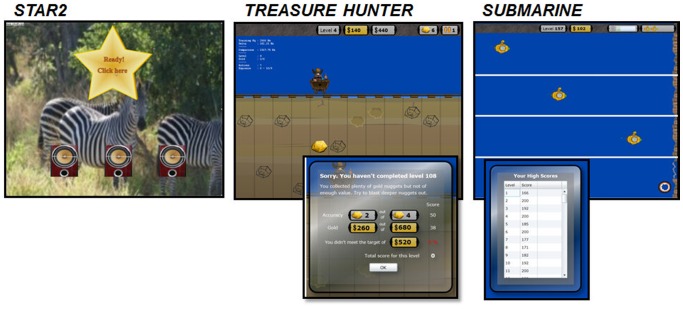
Screenshots of the three training platforms. *STAR2* background image rotates through a series of nature scenes unrelated to the task. *Treasure Hunter* is shown with an example of feedback after a level. *Submarine* is shown as successive snapshots to reflect movement across the screen, with an example of reward in the form of accrued points for each completed level.

Participants were loaned a laptop computer with a Yoga AD-200 USB Adaptor soundcard, and Sennheiser HD 25 headphones. After 20 minutes familiarization with the program in the research unit, training was performed by participants in their own home. Participants were randomly assigned to one of three groups: Group A started training with *STAR2*, Group B started with *Treasure Hunter*, and Group C started with *Submarine* (described below, see Figure 2 for screenshots). Participants were introduced to each training platform only at the point at which they began a period of training on that game. Participants were instructed to perform the training task for 30 minutes, five times a week for four weeks. They then crossed over to train on each of the other two games for one week each in succession (Fig. 1). Training duration, date, time, and performance throughout each training session was logged by the computer. The base sound level for training was fixed at 55 dB SL according to the better ear threshold measured at the training frequency. Level was roved at random within trials by ±6 dB SPL to remove loudness cues (c.f. [39]–[40]). Participants were trained on a single frequency standard within the normal-hearing range one octave below the audiometric edge, derived from their audiometric profile using a ‘broken-stick’ fitting procedure (125 Hz to 14 kHz) (c.f. [41]).

### Game development and beta testing with experienced tinnitus participants

Five participants who had previously taken part in a trial of FDT using STAR2 software [26] took part in beta testing of two newly developed games. They were observed in real training situations, and provided feedback on the usability and playability of each game. Participants were observed playing each game and completed a semi structured interview afterwards to discuss what they liked and disliked about each game. This was an iterative process to ensure consistency in how the games responded to the actions of the participant, check the clarity and consistency of information on game status, game instructions, and other visual information, and to implement changes to overcome or remove aspects of either game that were disliked. STAR2 software was not modified for the current study.

#### STAR2

*STAR* was developed at MRC Institute of Hearing Research as a platform for assessing frequency discrimination thresholds in children [42]. Training comprised a reactive task presented in a three-interval, three-alternate forced choice (3I-3AFC) ‘oddball’ discrimination paradigm, delivered as a continuous block of trials. For a given individual, the ‘trained’ fundamental frequency (the standard) was fixed throughout, and the target (the oddball) differed by percentage (Hz) above the fundamental frequency of the standard. An adaptive staircase procedure targeted performance at 79%. *STAR2* was a modified version of this test software for use in adults. Cartoon characters and backdrops were replaced with changing nature scene backdrops and picture images of a loudspeaker. *STAR2* indicates whether a trial has been successful or not immediately following the trial using different sound cues, but no other feedback or scoring system is provided. Training comprised a single level only so each new training session started at the same fundamental frequency difference between standard and oddball.

#### *Treasure Hunter*

*Treasure Hunter* was developed by co-author NVL. Training comprised of an interactive three interval task where the participant aims to progress through levels of the game by collecting sufficient reward points. Game play (selection, activation, and execution of frequency discrimination task) was participant induced. Participants had to move a mining cart left or right on the screen to ‘blast out’ gold nuggets buried in the ground, whilst avoiding coal nuggets. Gold nuggets had different levels of reward and nuggets buried deeper had greater reward. Coal nuggets had zero value. The ‘trained’ fundamental frequency was fixed throughout and the target always differed by a percentage (Hz) above the fundamental frequency of the standard; higher frequencies always corresponded to the presence of buried gold. The game had no time constraints, so participants could take a strategic approach to deciding which direction to move and when to select what they thought was a target. Rewards were earned by correctly identifying targets where the tone presented in the second interval was above the ‘trained’ fundamental frequency (i.e. presence of gold underneath). Participants were given a target value of reward to collect in order to progress to the next level of difficulty. Difficulty was increased in successive levels by reducing the percentage fundamental frequency difference that indicates reward. Feedback and scores were displayed after each level was attempted. Each new training session started at the level achieved in the previous session.

#### *Submarine*

*Submarine* was developed by co-author MSh. Training comprised an interactive two-interval task where the participant aims to progress through levels of the game by identifying hidden exits in the sea-wall. In contrast to *Treasure Hunter*, in this instance part of the game play was system induced because the submarine continuously travelled a horizontal path across the screen from left to right. Tones were presented as repeated pairs comprising a ‘sonar pulse’ from the submarine (set as the ‘trained’ fundamental frequency) followed by a response tone from a hidden gap to the right of the screen, such that the vertical point on the screen at which the tones were of identical frequency indicated the target (gap). The participant's task was to navigate the submarine up or down, raising or lowering the fundamental frequency of the second interval tone accordingly, to the point on the screen where the two tones were identical. This allowed the submarine to pass through the gap in the wall. Participants were given four ‘lives’ and had to pass through five gateways to progress to the next level of difficulty. Difficulty was increased on successive levels by reducing the possible percentage fundamental frequency difference between the two tones. Feedback and scores were displayed after each level was attempted. Each new training session started at the level achieved in the previous session.

### Assessment of intrinsic motivation – interview and thematic analysis

Participant experiences of FDT using each game were evaluated qualitatively using the methods described in Benedek and Miner [43]. In the first instance, participants viewed a set of 118 product reaction cards depicting positive, negative, and neutral descriptor words (Table S1). Participants selected all words they considered applicable to their experience of the training. They were then asked to select five words from all those selected that were most relevant, and to elaborate on their word choice in an interview that centred on these five words. Interviews were recorded and transcribed, and coded using a thematic analysis approach [44]–[45]. A protocol for the process was developed based on Braun & Clarke [46]. Transcripts were first read and reread to familiarize the researcher with the text. Sections of text that were identified as meaningful (codes) were selected independently by two of four co-authors (DHo, MS, SS, HA). The two researchers then met to agree on which constituted codes within each transcript. In a final stage three researchers independently considered whether each code related to one of four predefined themes related to intrinsic motivation; *challenge*, *control*, *fantasy*, and *curiosity* [32], or to other themes that were not pre-defined. Those codes related to intrinsic motivation were further categorised according to whether they coded for a (positive) motivating factor, or coded for a (negative) demotivating factor.

### Usability and game preference questionnaires

Usability of each game was assessed using a three item questionnaire asking (1) what did you like the most about the game, (2) what did you dislike the most about the game? and (3) what would you like to see changed to make the game better? At the end of the 6-week training period participants completed an overall evaluation questionnaire in which they ranked the three games in order of preference (1 =  liked most, 3 =  liked least) and provided written comments on their selection.

### Tinnitus handicap

Two questionnaires were used to measure self-reported tinnitus handicap. The Tinnitus Handicap Questionnaire (THQ) [47] is a validated measure of change in tinnitus severity with high test-retest repeatability [48]. Twenty-seven questions provide a global measure of tinnitus handicap (maximum score  = 2700). Scores >600 indicate tinnitus severity that disrupts daily activity [49]. The THQ has two reliable subscales: Subscale 1 - physical health, emotional and social consequences of tinnitus (15 questions), Subscale 2 - hearing difficulty (8 questions). The THQ was use as our primary outcome measure.

The Tinnitus Handicap Inventory (THI) [50] is a 25 self-report item questionnaire measure of tinnitus severity. Although some argue it is insensitive to change (e.g. [51]) it is widely used and has been reported as an outcome in studies of FDT for tinnitus [23] [52]. Test-retest reliability of the THI is high [53].

Questionnaires were administered at screening, T0, T1, T2, and T3.

### Psychoacoustic measures of tinnitus

Baseline tinnitus quality and changes over time were measured using the Tinnitus Tester [54], an automated computerised assessment of the qualities of the tinnitus sensation (matched loudness, dominant pitch, and bandwidth) over a 0.5–12 kHz frequency range. In addition to rating tinnitus loudness on a visual analogue scale (VAS) participants matched loudness by adjusting the level of a range of sound clips (centre frequencies 0.5–12 kHz) until each was judged to be the same as their tinnitus level. We took the loudness measure as the matched value at a single-frequency corresponding to little or no hearing loss (typically 0.5 or 1 kHz) and distant from the dominant tinnitus pitch. A profile of the individual tinnitus spectrum was generated by asking participants to rate the likeness of 11 sounds (centre frequencies 0.5–12 kHz) to the pitch of their tinnitus, using a 100-point scale. The dominant tinnitus pitch is defined as that frequency in the spectrum which had the highest likeness rating. Bandwidth was calculated as the standard deviation of all frequencies in the tinnitus spectrum, where each frequency was weighted by its percentage likeness to the tinnitus pitch of the participant (c.f. [41]). To reduce the impact of procedural learning on tinnitus outcomes the Tinnitus Tester was administered twice before training with the second measure taken as baseline (T0).

### Test of Everyday Attention (TEA)

The Test of Everyday Attention (TEA) is a standardised clinical test battery that allows for comparison across different attentional capacities in adults [55]. Two subtests were completed. Subtest 6 is a speeded visual task which measures selective attention. Participants are asked to search for and mark pairs of symbols from a list of entries in a simulated classified telephone directory (using list version A at baseline). The telephone search with counting (Subtest 7) measures divided attention by asking participants to perform the same speeded visual task (using a different version A list) whilst simultaneously counting tones presented from a loudspeaker and recalling the number of tones when prompted. Performance on the individual tasks was calculated as average time per item. Dual task detriment was then calculated as the increase in time per item required in the divided attention task compared to the sustained attention task.

The presentation level of the tone was sufficient to be clearly audible to the participant. Participants were assessed at baseline (T0) and again four weeks later (T1) when training on the first game was completed. Version B of each test was administered at T1.

### Analysis of quantitative data

For THQ and THI scores (measured at four time points) 7.9% of values were missing. For the TEA (measured at two time points) 6.7% of values were missing. These missing values were imputed using an expectation-maximisation (multiple imputation) method which assumes a normal distribution for the partially missing data and bases inferences on the likelihood under that distribution (maximum 25 iterations, SPSS v16.0).

Main analyses were conducted using analysis of variance models that included significant covariates to account for the influence of potential confounding factors (age, audiometric threshold, baseline depression and anxiety). Covariates for inclusion in the model were determined from initial analyses which included all potential covariates. Our primary analyses were (1) evidence that gameplay made FDT intrinsically motivating, (2) change in tinnitus handicap and in performance on attention tasks between T0 (baseline) and T1 (after training on the first game). Secondary analyses looked at effects across the multiple time points of this study. Clinical effect sizes were calculated as partial eta-squared (ηp^2^) on account of the repeated-measures design [56].

## Results

### Recruitment details

Recruitment began on 23^rd^ July 2011 and the final follow-up assessment was completed 30^th^ August 2012.

### Baseline characteristics

Table 1 shows mean baseline characteristics of each group. All three groups had a mean baseline tinnitus handicap sufficient to disrupt daily activity (i.e. >600; [43]).

**Table 1:** Baseline characteristics and training details.

	Group A	Group B	Group C
**Measure**	Mean (SD)	Mean (SD)	Mean (SD)
**Gender**	12 M, 8 F	12 M, 8 F	10 M, 10 F
**Age**	60.2 (12.5)	57.8 (14.0)	60.6 (11.4)
**PTA (dB HL)**	31.8 (17.1)	25.2 (17.6)	32.2 (18.0)
**Hearing loss slope**	5 gradual, 15 steep	5 gradual, 15 steep	11 gradual, 9 steep
**Tinnitus duration (years)**	12.6 (11.9)	10 (9.7)	11.4 (11.2)
**Depression**	6.9 (8.3)	5.2 (3.6)	6.2 (8.1)
**Anxiety**	7.7 (7.1)	8.0 (7.1)	5.8 (4.7)
**Hyperacusis**	12.3 (7.5)	12.1 (5.9)	13.1 (7.3)
**Global THQ (/2700)**	906 (485)	937 (452)	1040 (440)
**THQ Subscale 1 (/1500)**	449 (323)	467 (291)	481 (315)
**THQ Subscale 2 (/800)**	278 (176)	289 (178)	366 (183)
**VAS loudness (/100)**	46 (20.8)	39.8 (15.9)	38.6 (14.9)
**Sensation level (dB SL)**	29 (16)	25 (16)	17 (11)
**Dominant pitch (kHz)**	7.6 (3.0)	6.1 (2.7)	6.4 (3.6)
**Tinnitus bandwidth (units)**	3 (0.7)	3.1 (0.6)	3.3 (0.4)
**Training frequency (kHz)**	0.9 (0.7)	1.4 (1.1)	1.3 (1.2)

PTA: Pure Tone Average calculated as the average hearing threshold for 0.5, 1, 2 and 4 kHz, averaged across both ears. THQ: Tinnitus Handicap Questionnaire. VAS: Visual Analogue Scale.

### Compliance

Compliance with the initial block of training was calculated as a percentage of the time on task that had been prescribed (i.e. 30 min×5/week×4 weeks). For Group A *(STAR2)* compliance was 69% (SD = 21), for Group B (*Treasure Hunter*) it was 67% (SD = 22), and for Group C (*Submarine*) compliance was 75% (SD = 24). Compliance did not differ significantly between groups.

### Outcomes

#### 1. Motivation and preferences

**Figure 3:**
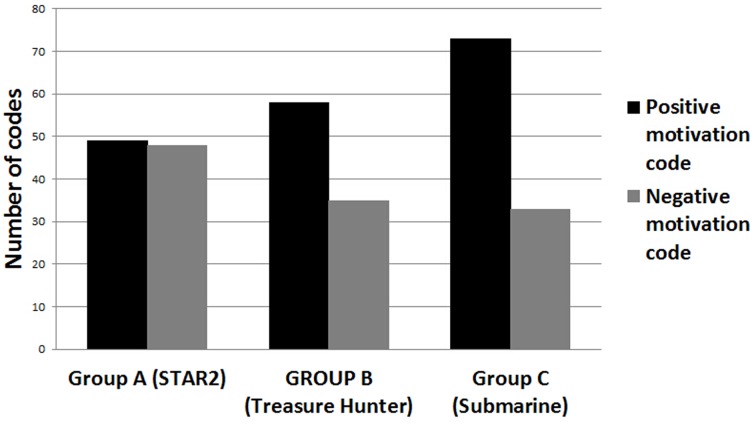
Frequency of codes related to intrinsic motivation. Data were extracted from 55 interviews in total (n = 18 for *STAR2*, 18 for *Treasure Hunter*, and 19 for *Submarine*).

Motivations relating to participation and compliance were evaluated as part of a qualitative post-training assessment. 55 participants returned for their first post-training interview at T3. Analysis of these interviews generated 371 codes. Extrinsic motivators such as potential for improvements in tinnitus and a commitment to take part in the research were mentioned by comparable numbers of participants across all three groups but were not discussed further. We judged that 294 of the initial 371 codes related to one of the four themes under intrinsic motivation (challenge, control, fantasy, or curiosity). The pattern of reporting differed significantly across groups (*X* ^2^ = 7.296, *p* = 0.026, Figure 3). For *STAR2* there was a similar number of positive and negative codes related to intrinsic motivation (49 and 48 codes respectively), for *Treasure Hunter* there were more positive than negative codes (48 compared to 35), and for *Submarine* the majority of codes were positive (73 compared to 33 negative).

The most frequent positive codes were associated with the *Submarine* game and were related to the theme of ‘control’ (42 codes, Table 2). Participants felt *Submarine* was understandable and straightforward to use, which supported ‘a desire to beat the game’. Most frequent negative codes were associated with *STAR2* and were related to the theme of ‘curiosity’ (33 codes, Table 2); participants felt that training on STAR2 was repetitive and boring, and that the training period (30 minutes) was too long.

**Table 2:** Number of codes per theme.

	Code Valence	STAR2	TREASURE HUNTER	SUBMARINE
**Challenge**	Positive	18	23	23
	Negative	13	9	6
**Control**	Positive	24	25	42
	Negative	2	7	2
**Fantasy**	Positive	0	2	1
	Negative	0	2	3
**Curiosity**	Positive	7	6	7
	Negative	33	17	22

Usability and game preference was determined by a brief questionnaire. Of those who had experience of all three games (n = 54), most (n = 26) expressed a preference for *Submarine*. *Treasure Hunter* was preferred by 23 participants and just five ranked *STAR2* as their preferred game. Written text provided by participants on usability and overall evaluation revealed that those who preferred *STAR2* did so because of its ease of use, finding the other games difficult or frustrating to play. For those who preferred *Treasure Hunter* or *Submarine* however, the major theme was that these games provided a sense of reward or achievement and were challenging, engaging, and stimulating.

#### 2. Tinnitus

**Figure 4:**
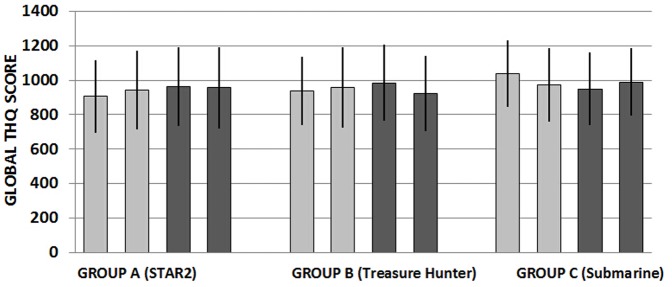
Global Tinnitus Handicap Questionnaire scores. Mean tinnitus handicap score (±95% CI) at the primary assessment points (T0 and T1 – black bars) and at follow up visits T2 and T3 (grey bars). n = 20 per group. Global score range in 0–2700.

Our primary tinnitus outcome was change in THQ score (global measure of tinnitus handicap) at T1 compared to baseline (T0). Mean global THQ scores are given in Figure 4. Whereas mean THQ score increased for Group A (by 26 points) and Group B (by 20 points), for Group C THQ score was reduced by 69 points. A mixed design ANOVA was conducted with the within-subject factor of time (T0, T1) and the between-subject factor of training regime (*STAR2*, *Treasure Hunter*, or *Submarine*), with hearing loss included in the model as a significant covariate. Within-subject tests revealed a statistically significant change between T0 and T1 [*F* (1,56) = 5.956, MSE = 117909.794, *p* = 0.018, *ηp* ^2^ = .096]. Although a medium effect size, the difference in change between groups was less than is assumed to be clinically meaningful (194 points). There was no significant effect of training regime or interaction between time and training regime (p>0.05). Hence, we found no evidence that the type of gameplay modulates change in tinnitus handicap. Analyses of overall THQ subscale scores showed a small statistically significant effect [*F* (1,56) = 5.931, *MSE* = 208.387, *p* = 0.047, *ηp^2^* = .047] for THQ subscale 1 (health and psychological wellbeing) but not for THQ subscale 2 (hearing difficulties) (*p*>0.05) indicating the overall change related to change in psychological rather than functional handicap.

In all cases the main effects above were not maintained at T3 follow-up (compared to T0 *p*>0.05 in all cases). Analysis of the THI scores revealed non-significant results, indicative of the lack of sensitivity to change of this questionnaire measure [44].

A mixed design ANOVA showed that after 4 weeks training (T1 compared to baseline T0) there was no significant effect of time or training regime on VAS loudness scores (*p*>0.05 in all cases), and no significant interaction of the main factors. Psychoacoustic measures of tinnitus quality include loudness rating, dominant tinnitus pitch, and bandwidth. For matched tinnitus loudness there was also no effect of time. There was however a significant effect of training regime [*F* (2,57) = 3.255, MSE = 1281.258, *p* = 0.046], but no significant interaction of time and training regime. Pairwise comparisons were significant for a difference between Group A and Group C only (*p* = 0.014), where mean matched loudness level did not change, and increased by 3 dB SPL respectively. There was also no correlation between change in tinnitus loudness and change in tinnitus handicap however (*r* = −0.087, *p* = 0.51). Using the same approach to analysis, we found no significant effect of time or training regime on dominant tinnitus pitch or tinnitus bandwidth (*p*>0.05 in all cases). Neither were there significant interaction effects. As for tinnitus handicap, our results show that the type of gameplay has no impact on tinnitus quality.

#### 3. Attention

A mixed-design ANOVA was used to assess the effect of training on attention. Age was included in the analysis as a significant covariate. Effects of the within-subject factor of time (T0, T1) and the between-subject factor of training regime (trained on *STAR2*, *Treasure Hunter*, or *Submarine*) were modelled.

Sustained attention (speeded visual search): There were no significant effects of time, training regime, or significant interaction (p>0.05).

**Figure 5:**
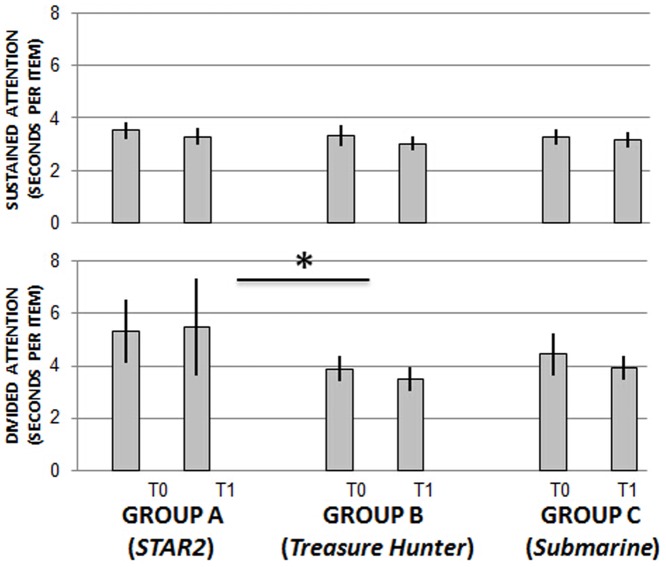
Sustained and divided attention task scores before and after training. There was no significant change in the measure of sustained attention after training. There was a significant between groups difference on the divided attention task for Group A compared to Group B but there was no effect of time or interaction (*p<0.05). n = 20 per group.

Divided attention (speeded visual search while counting): There was no significant effect of time on divided attention (*p*>0.05) (Figure 5). There was a significant effect of training regime [*F*(2,57) = 3.946, MSE = 31.27, *p* = 0.025, *ηp^2^* = .122]. Pairwise comparisons showed that the significance related to Group A (*STAR2*) and Group B (*Treasure Hunter*) (*p* = 0.033 after bonferroni correction), reflecting the overall differences in scores of these two groups. There was no significant interaction between time and training regime on divided attention (*p*>0.05). Hence we found no evidence that the type of gameplay affects changes in performance on attention tasks.

Dual task detriment: As expected from the results above, there was no overall effect of time or significant interaction (*p*>0.05) but there was a significant effect of training regime on dual task detriment [*F*(2,56) = 3.841, MSE = 23.651, *p* = 0.027, *ηp^2^* = .121]. Pairwise comparisons again showed that the significance was between Group A (*STAR2*) and Group B (*Treasure Hunter*) (*p* = 0.028 after bonferroni correction).

This is the first study to examine the effects on FDT for tinnitus on attention. As the result was contrary to our hypothesis further analyses were conducted to investigate whether there had been any relationship between baseline tinnitus severity and performance on the attention tasks. No correlation was found between baseline tinnitus severity and either sustained or divided attention (*r* = 0.04, *p* = 0.764 and *r* = 0.033, *p* = 0.8, respectively). Partial correlation to factor in differences in audiometric threshold had no effect on the relationship (*r* = −0.12, *p* = 0.93 for sustained attention and *r* = −0.13, *p* = 0.92 for divided attention). However, audiometric threshold did correlate significantly with sustained attention (*r* = 0.27, *p* = 0.037), and approached significance for divided attention (*r* = 0.244, *p* = 0.061). So whilst there was no evidence that different levels of tinnitus severity impact on attention, at baseline, better audiometric threshold was associated with better performance on these tests. Hence, we speculate that hearing loss negatively impacts cognitive performance.

## Discussion

This study addresses two questions. First, can gameplay make FDT more rewarding and intrinsically motivating? Second, would elements of intrinsic motivation that are inherent in game-play usefully increase the benefit a person with tinnitus gets from doing FDT (reduce tinnitus handicap, improve cognitive performance)?

### Intrinsic motivation in FDT

We conducted a qualitative evaluation to understand and compare intrinsic motivations to perform FDT where the FD task is delivered as a simple reactive task (as used in psychophysical testing), or where the FD task is integrated with progressing through levels of a computer game. Analysis of semi-structured interview data clearly showed that greater intrinsic motivation was associated with the gameplay on *Treasure Hunter* and particularly on *Submarine*, than with the simpler reactive task-based training of *STAR2*. Most participants reported a preference for FDT on *Treasure Hunter* and *Submarine*. Despite differences in motivation and game preference, compliance with prescribed training was similar across all groups. The intrinsic motivations identified were in any case insufficient to promote high compliance. We conclude that, whereas intrinsic motivation through gameplay may promote enjoyment and compliance with interventions in other health or educational domains (e.g. [57]–[58], in the case of this population (people with tinnitus) and this intervention (FDT), it promotes enjoyment but has no effect on compliance.

### Effects of training on tinnitus

Our previous work showed that the benefit derived from FDT in terms of a small reduction in tinnitus handicap was independent of the training frequency chosen and not related to a change in tinnitus quality [26]. Here again we find a small effect of FDT on our primary measure of tinnitus handicap, but within groups, mean THQ score only changes by up to 69 points (∼7% of baseline) after training (T1) and there was no difference between groups.

### Effects of training on measures of attention

We also took the opportunity here to test the effect of FDT on attention in a tinnitus population. A number of studies point to deficits in cognitive processes such as attention and working memory in people with tinnitus, and for more cognitively demanding tasks in particular [59]–[62]. So, if FDT does improve attention then it may be useful for tinnitus. Training may reduce the amount of attention given over to the tinnitus sound and thereby increase the attentional capacity available to better perform everyday activities. Indeed, redirecting attention through movement therapy has already been applied clinically within a multi-therapy approach to tinnitus management [63].

Detrimental effects of tinnitus on measures of attention have previously been demonstrated in sub-populations of people with clinically significant tinnitus. Physiological effects were demonstrated by Delb et al. [64] who showed that tinnitus distress level impacts on the attention effects on event-related potentials (N100, phase locking), concluding that attention resources are ‘captured’ by tinnitus in people with higher distress levels. Rossiter et al. [61] showed there were slower reaction times on a dual task in people with moderately bothersome tinnitus compared to non-tinnitus controls. In a follow-up study Stevens et al. [62] observed slower reaction times in people with severe tinnitus in both a Stroop paradigm and a divided attention task; higher self-reported tinnitus handicap was associated with slower reaction times. Hallam et al. [59] also found that people with tinnitus show slower reaction times in a dual task condition compared to no tinnitus controls.

Here we observed no significant effect of FDT on the performance of a sustained or divided attention task. Furthermore, our results suggest that whereas the degree of hearing loss might determine performance on tasks of attention, there is no indication the degree of tinnitus handicap affects how well they perform these tasks. This is particularly contrary to the findings of Stevens et al. [62] who compared a small sample of tinnitus participants (n = 11 compared to 60 here) to a non-tinnitus control group. Stevens et al. [62] only included the degree of hearing loss at high frequencies (the average audiometric threshold for 4,6,8 kHz) in their analysis of covariance, rather than loss at the lower frequencies more relevant to speech and music perception (up to 2 kHz).

These secondary observations point to future work to fully understand the degree to which tinnitus and hearing loss contribute to performance on cognitive tasks.

### Limitations of this study

As with many studies of FDT for tinnitus, one limiting factor is the short training period prescribed and completed. The overall effect on THQ score in this study fell short of that reported in our previous study [26]. At our primary endpoint (after 4 weeks training) compliance was ∼20% less than that observed in our previous study. We can speculate that this was related to factors such as (1) a longer intervention period in the current study (6 weeks compared to 4 weeks), (2) a much larger team on the current study with less continuity of the relationship between assessor and participant, or (3) ceiling effects in the previous study. Greater compliance here may have given an equal or greater effect on THQ score to that seen previously but even with identifiable extrinsic and intrinsic motivators, compliance looks likely to remain an issue for this particular intervention and participant group.

Another potential limitation, in terms of our results on the effects of attention, is that the outcome measure we chose (TEA) may not appropriately capture the changes in attentional processing that may occur as a result of FDT. A single measure of sustained attention for example may be insufficient to support or refute a main effect [65].

### Future directions

Perhaps the more challenging indications to emerge from this study are those relating the performance on attention tasks to the degree of hearing loss, and not tinnitus severity. Previous studies might suggest that cognitive training should be explored as a plausible therapeutic avenue. Indeed, psychotherapeutic approaches to tinnitus management already include elements termed attention training [66] or attention redirection [63]. Computer-based approaches to training attention, if sufficiently intrinsically motivating, may serve as an alternative self-help tool for people with tinnitus. However there is clearly first a need to explore the true impact of tinnitus on cognitive resources. The limited participant numbers and lack of control for hearing loss across previous studies on the topic mean further studies are needed to disambiguate the effect of tinnitus on cognitive performance from the effects of hearing loss or other factors. Are there subsets of tinnitus patients who show particular deficits on attention demanding tasks? If so then this may be a route for targeted management.

## Conclusion

FDT for tinnitus has now been the topic of eight published studies which taken together suggest it has a reproducible but small effect on tinnitus handicap more likely to be due to a change in cognitive representations (e.g. emotional reaction) rather than a physiological change in the auditory system (e.g. hearing) [19] [26]. For most people, delivering FDT in a way that uses standard game-play approaches to intrinsically motivate the ‘player’ is preferred to a simple task-based training regime but this does not in itself lead to compliance or to additional improvements in self-reported tinnitus severity. The results of this study, taken in context with the limited existing literature, suggest that cognitive deficits experienced by people with tinnitus can be improved through training, if training incorporates the right intrinsically motivating elements and engages the user. It remains to be investigated whether such improvements can lead to clinically important improvements in tinnitus handicap.

## Supporting Information

Checklist S1 **CONSORT Checklist.**
(DOC)

Protocol S1 **Trial Protocol.**
(PDF)

Table S1 **Codes on product reaction cards.**
(TIF)
