# Supplementary material for: Gameplay as a Source of Intrinsic Motivation in a Randomized Controlled Trial of Auditory Training for Tinnitus
Source: PLoS One. 2014 Sep 12;9(9):e107430. doi: 10.1371/journal.pone.0107430 (PMC4162598; doi:10.1371/journal.pone.0107430)
Supplement: Protocol S1 — Trial Protocol. (PDF) [file pone.0107430.s002.pdf]

Auditory Games for Tinnitus Benefit

Study protocol

1 Research Organisation

NIHR National Biomedical Research Unit in Hearing

2 Research Sponsor

Nottingham University Hospitals NHS Trust

3 Research Team

(Chief Investigator) Prof Deborah Hall
NIHR National Biomedical Research Unit in Hearing, Ropewalk House, 113 The Ropewalk, Nottingham, NG1 5DU
Tel: 0115 8232644
Email deb.hall@nottingham.ac.uk

(Study Co-ordinator) Dr Derek Hoare
NIHR National Biomedical Research Unit in Hearing, Ropewalk House, 113 The Ropewalk, Nottingham, NG1 5DU
Tel: 0115 8232630
Email derek.hoare@nottingham.ac.uk

(Advisory collaborator) Prof Mike Sharples
Learning Sciences Research Institute, University of Nottingham Jubilee Campus, Wollaton Road Nottingham, NG8 1BB
Tel: 0 115 9513716
Email Mike.sharples@nottingham.ac.uk

(Co-investigator) Dr Nicolas van Labeke
[1]Learning Sciences Research Institute, University of Nottingham Jubilee Campus, Wollaton Road Nottingham, NG8 1BB
[2]NIHR National Biomedical Research Unit in Hearing, Ropewalk House, 113 The Ropewalk, Nottingham, NG1 5DU
Tel: (0115) 846 6561
Email: nicolas.vanlabeke@nottingham.ac.uk

(Research Associate) Miss Victoria Kowalkowski
NIHR National Biomedical Research Unit in Hearing, Ropewalk House, 113 The Ropewalk, Nottingham, NG1 5DU
Tel: (0115) 8232642
Email: vistoria.kowalkowski@nottingham.ac.uk

(Statistician) Miss Sujin Kang
NIHR National Biomedical Research Unit in Hearing, Ropewalk House, 113 The Ropewalk, Nottingham, NG1 5DU
Tel: 0115 8232631
Email: sujin.kang@nottingham.ac.uk

All team members who will have contact with participants will have an honorary contract with Nottingham University Hospitals Trust.

4 Synopsis

Title	**Auditory Games for Tinnitus Benefit: Interactive versus Reactive Auditory Discrimination Games**
Acronym	TinGames
Chief Investigator	Prof. Deborah Hall
Objectives	To investigate whether interactive auditory games have additional benefit over reactive auditory games for tinnitus.
Study design	Single-centre crossover comparison, between groups design.
Setting	Audiological and tinnitus testing will take place at NBRUH.
Sample size estimate	Power analyses using simulation were performed (SAS® software version 9.2) using data from a comparable study for between group mean comparisons of Tinnitus Handicap Questionnaire.
Number of participants	It was estimated that at least 20 participants per group would be required for significance in a one-sided test at alpha 0.05. 27 participants per group would be required to detect a large effect size difference between two group means (to achieve a power of 0.8 for a two-sided test at alpha 0.05). We will therefore recruit at least 20 participants to each group and up to 27 participants per group including any participants who drop out.
Eligibility criteria	Adults with chronic tinnitus not currently availing of other treatment for tinnitus or related condition.

Description of interventions	Participants will complete questionnaires, undergo standard audiological and tinnitus testing, and train on a simple auditory training game for 15-30 minutes per day, 5 days per week for 6 weeks.
Duration of study	On receiving REC and R&D approval participant recruitment will begin immediately, and continue until the sample size is met.
Statistical methods	Comparisons will be drawn within and between groups with respect to the primary and secondary outcome measures below using Paired t test/McNemar's test, t test, ANOVA/Kruskal–Wallis test and Linear regression/the General Linear Model/the Generalized Linear approaches. Secondary analyses will adjust for the factors, e.g. initial severity of tinnitus, type of hearing loss, age and gender, as appropriate.

5 Funding

The research is fully funded as part of a National Institute for Health Research (NIHR) Biomedical Research Unit. The current grant is awarded until the end of March 2012.

6 Project dates

April 2010 – March 2012

7 Background Information and Rationale

Tinnitus refers to a person's perception of a ringing, hissing or buzzing sound despite there being no such sound in the external world. It affects 10-15% of the population in the UK (Davis & El Rafaie, 2000). Despite tinnitus being so widespread it is poorly understood and there is no uniformly effective treatment. The exploration of novel treatments for tinnitus is welcome, especially if these treatments are effective and reduce the cost of current service provision (Aazh et al., 2009, Newman et al., 2008). Recent advances in the understanding of changes in the central auditory system (e.g. with the use of neuro-imaging) and their relation to tinnitus perception, has led to a focus on the effect of cortical re-organisation and to suggestions that forms of active auditory training, based on narrow-band sounds, might provide effective techniques for tinnitus management (Mühlnickel et al., 1998, Eggermont & Roberts, 2004, Herraiz et al., 2010).

The motivation for auditory training arises from the potential that active listening tasks can be tailored to correct the over-representation of particular frequencies in the cortex. Specific training should result in an expansion of the trained frequencies and shrinking of adjacent zones. We recently published a systematic review of the evidence that auditory training may have benefit for people with tinnitus (Hoare et al., 2010). Overall, the evidence is positive but clearly not yet robust enough to provide confidence that auditory training is a viable management technique. However, our current Randomised Controlled Trial of Frequency Discrimination Training (approved by Derbyshire REC in November 2009) is providing evidence

that training at pure tone frequencies, for which the participant has clinically normal hearing, has more benefit in terms of reduced tinnitus intrusiveness, than training at frequencies (pure tones or harmonic complexes) where the participant has some level of hearing loss. Our next challenge therefore is to build on this finding in ways that might maximise the benefits we observe.

The training software we currently use (STAR2) was originally developed by the Institute of Hearing Research, Nottingham, for use with children. Our past participants have given mixed reviews of this software, some reportedly enjoying the training, some finding it soporific. Others however, reported that it is monotonous and un-motivating. We therefore wish to explore the impact of different game mechanics in the delivery of auditory discrimination training and have designed two different interactive games in the context of training for tinnitus benefit. Specifically, we wanted to generate games that would deliver the same type of auditory training as the software we currently use, but would be intrinsically motivating, i.e. be a game that the participant is motivated to play irrespective of any potential benefit for tinnitus. Our current game (STAR2) can be described as reactive, i.e. the sounds play and the participant selects what they think is the correct answer (odd-one-out). The two new games we wish to test can be described as interactive, i.e. participants control the sound delivery and actively seek the correct answer: this may have additional benefit for tinnitus.

References

- Aazh H, Moore BCJ, Roberts P (2009) Patient-centered tinnitus management tool: A clinical audit. *American Journal of Audiology*, 18, 7-13
- Davis A, El Rafaie A (2000) Epidemiology of tinnitus; in *Tinnitus Handbook*. San Diego, Singular Publishing Group, pp 1-23
- Eggermont JJ., & Roberts LE. (2004) The neuroscience of tinnitus. *Trends in Neurosciences*, 27, 676-682
- Herraiz C, Diges I, Cobo P, & Aparicio JM (2009) Cortical reorganisation and tinnitus: principles of auditory discrimination training for tinnitus management. *European Archives of Oto-Rhino-Laryngology*, 266, 9-16
- Hoare DJ, Stacey PC, & Hall DA (2010) The efficacy of auditory perceptual training for tinnitus: A systematic review. *Annals of Behavioral Medicine*. 40:313–324
- Mühlnickel W, Elbert T, Taub E, & Flor H (1998) Reorganization of auditory cortex in tinnitus. *Proceedings of the National Academy of Sciences of the United States of America*, 95, 10340-10343
- Newman CW, Sandridge SA, Meit SS, Cherian N (2008) Strategies for managing patients with tinnitus: A clinical pathway model. *Seminars in Hearing.* 2008, 300-309

8 Objectives and Purpose

Purpose: This research project will explore whether auditory discrimination training delivered as interactive games has significant benefit over training delivered in a reactive game (STAR2) format.

Primary objectives:

- Do interactive games have more benefit than reactive games as auditory training for tinnitus?
- Are interactive games more usable and desirable as forms of auditory training for tinnitus?

Secondary objectives

- Is auditory training for tinnitus indicated for particular subgroups of tinnitus patients (e.g. who have steep-sloping hearing loss, high frequency tinnitus, or have had tinnitus for shorter time-periods)?
- Does auditory discrimination training provide a purely acoustic benefit or are the changes in attention also important in determining the participant's improvement?

9 Study design

Single-centre, between-subjects design with minimized allocation to different training groups. The primary endpoints are the pre- and post- training measures that provide the primary measures of efficacy. These include the audiological measures and questionnaire responses about thoughts and feelings associated with tinnitus, generic measures of attention, and measures of user acceptability. All assessments will take place at NBRUH. Training will take place in the participants own home using a loan-laptop configured to deliver the training program and record progress and compliance.

10 Selection and Withdrawal of Participants

10.1Recruitment of Participants

We currently have a department wide system of recruiting participants through leaflets placed in Nottingham Audiology and ENT departments. Potential participants identify themselves to NBRUH and are contacted by our central recruitment co-ordinator who assesses their essential suitability and provides them with details of the relevant studies. Participants can then contact the study co-ordinator directly. We currently have a significant and ever growing database of people with tinnitus who are interest in participating in our research.

We more broadly advertise our research studies to the general public. Three examples include the NBRUH website, interviews with the local newspapers, radio, and TV, and articles for appropriate charity magazines (Deafness Research UK and the British Tinnitus Association).

10.2 Inclusion Criteria

(i) Chronic subjective tinnitus (experienced for over 6 months)
(ii) Aged 18 + years old
(iii) Not currently receiving treatment for tinnitus from the NHS or other sources

10.3 Exclusion Criteria

(i) Significant distress (Beck anxiety score >25, Beck depression score >13)
(ii) Hyperacusis (Khalfa Hyperacusis Questionnaire score >27)

(iii) Significant bilateral hearing loss (>39 dB at all tested frequencies)

10.4 Expected Duration of Participant Involvement

The study requires a commitment to complete between 7.5 and 15 hours of auditory training over a period of 6 weeks. The duration of training to be completed will be informed by our current studies (i.e. we will use the duration of training which provides maximum benefit). Participants will be asked to attend 6 assessments (Initial assessment will take 2 hours, subsequent assessment will take 1-1 ½ hours) over a period of 12 weeks. All assessments will take place at NBRUH.

Participants will receive an inconvenience payment of £5 per hour (or part thereof) up to a maximum of £10 for attending each assessment session at NBRUH, and reimbursement of travel expenses incurred up to a maximum of £15 (petrol will be paid at 20p per mile). The training period will not attract any payment, however further incentive to complete the study will take the form of a prize draw for a £50 John Lewis voucher.

10.5Informed Consent

All participants will receive a participant information sheet before their first appointment at NBRUH. The information sheet explains the aims of the study, the experimental procedures, and other useful information, such as who will see the data and what happens if someone wishes to make a complaint. Participants will also receive a consent form explaining their rights and responsibilities. The consent form clearly states that the participant is free to withdraw at any time without any need to give a reason and without affecting future medical care. At the assessment appointment, the consent form will be signed by both the researcher and the participant. A signed copy will be given to the participant.

Dr Hoare will provide participants with any clarification or additional information pertaining to taking part in the study. We do not have access to translator services and the consent forms and information sheets will not be available in other languages other than English. It will be explained to the potential participant that their participation in the study is entirely voluntary and that their medical treatment and care will not be affected by their decision. It will also be explained to participants that they can withdraw at any time without giving any reason. In the event of a participant withdrawing it will be explained that their data collected to that point cannot be erased and we will seek consent to use the data in the final analyses where appropriate.

10.6Allocation of Participants into Training Groups

Participants will be randomized to groups using minimisation (Altman & Bland, 2005) to ensure the groups are balanced with respect to (i) severity of tinnitus; Tinnitus Handicap Questionnaire score; <600, 600-1200, or >1200, (iii) age; 18-50, 50-70 *or* 70+ (categories based on RNID statistics* for moderate to severe hearing loss), and (iv) sex.

The member of the study team that enrols the participant and completes the pre-study assessments will not be same person who assigns participants to groups. The post-study tinnitus assessments will be carried out blind by a researcher who will not know which group the participant was allocated to. All participants will receive the same information about the expected treatment outcomes.

- Altman DG, Bland JM (2005) Treatment allocation by minimisation. *British Medical Journal* 330, 384

- *http://www.rnid.org.uk/information_resources/aboutdeafness/statistics/statistics.htm#age

10.7 Removal of Participants from Training

A participant will be withdrawn from the study if they commence any other form of tinnitus treatment. Training will be discontinued if the participant reports an adverse event. The definition of an adverse event in the context of this project is (1) auditory discrimination training increases tinnitus intrusiveness and makes it unbearable, (2) a disease or symptoms (unrelated to tinnitus) that were present at baseline and that worsened following the start of the study.

11 Measurements

The pre- and post- treatment measures provide the primary and secondary measures of change in tinnitus and efficacy. These include the acoustic measures and questionnaire responses about thoughts and feelings associated with tinnitus, and generic measures of everyday attention. Participants will complete two assessments before commencing training to account for the learning effects inherent in our test battery.

The initial screening assessment will involve

- Tinnitus Case History Questionnaire (TCHQ) – self reported tinnitus, general health and lifestyle questionnaire to collect baseline characteristics of participants
- Hyperacusis Assessment Questionnaire – a questionnaire to assess hypersensitivity to certain sounds.
- Emotional state - Standardised self-reported measure of depression and anxiety (Beck's questionnaires – BAI, BDI)
- Tinnitus Handicap Inventory (THI) – A questionnaire to categorise the severity of a person's tinnitus.
- Tinnitus Handicap Questionnaire (THQ) – a questionnaire that is sensitive to changes in a tinnitus intrusiveness, suitable to measure change over time.
- Pure Tone Audiometry - A listening test that measures hearing ability up to 16 kHz
- Tinnitus Tester - A listening test that establishes a match to the pitch and loudness of the tinnitus percept
- Test of Everyday Attention (TEA) – Subtest 6 and 7 of the validated TEA test battery will be used to measure the ability to sustain attention on a task (Test of Sustained

Attention), and measure difficulty when undertaking 2 tasks at the same time (Dual Task Detriment).

All subsequent assessments will involve the

- THQ
- THI
- Tinnitus Tester
- TEA

Post-training assessments will also include

- Pure-tone Audiometry (once at the end of training to ensure no change in hearing has occurred)
- Usability Questionnaire (after 4, 5 and 6 weeks training). This is a short questionnaire with free text responses to the questions: What did you like most about the game? What did you dislike most about the game? What would you like to see changed to make the game better?
- Desirability Questionnaire (after 4, 5 and 6 weeks training) (Benedek & Miner, 2002). This requires participants to select words from on 118 'product reaction cards' that indicate their attitudes towards the game experience. For the five words indicated as most appropriate, the participant then completes a short free test questionnaire to give reasons for the choice.
- Overall evaluation (after 6 weeks training). Participants are asked to rank the three games in order of personal preference and indicate the reasons for their preference.

The THQ will be the primary measure of training efficacy as it is used and well documented in peer-reviewed research studies (Bauer and Brozoski, 2006; Henry et al, 2006; Londero et al, 2006) involving patient groups similar to those proposed here.

- Bauer CA, Brozoski TJ (2006) Effect of gabapentin on the sensation and impact of tinnitus. *The Laryngoscope,* 116, 675-681
- Benedek, J., Miner, T., 2002. Measuring desirability: new methods for evaluating desirability in a usability lab setting. In: Proceedings of Usability Professionals Association, Orlando, July 8-12.
- Henry JA, Schechter MA, Zaugg TL, Greist S, Jastreboff PJ, Vernon JA, Kaelin C, Meikle MB, Lyons KS, Stewart BJ (2006) Clinical trial to compare tinnitus masking and tinnitus retraining therapy *Acta Oto-Laryngologica,* 126, 64-69
- Londero A, Peignard P, Malinvaud D, Avan P, Bonfils P (2006) Tinnitus and cognitive-behavioral therapy: results after 1 year. *La Presse Medicale,* 35, 1213-1231

12 Training Regimen

Each auditory discrimination training session will involve performing the same listening task. For **Game A** (STAR2) participants are required to indicate which sound is the 'odd one out' in each set of three sounds. Over time, the task will get harder according to individual

performance (the perceptual difference defining the odd one out gets smaller as the participant responds correctly and vice-versa). In **Game B** (Submarine), the auditory discrimination task is built into "sonar bursts". Participants have to navigate the submarine in order to match the two signals. In **Game C** (Metal Detector), game-play is decided solely by the interaction of the participant, to which the system responds by generating the appropriate auditory signals. Participants have to locate 'gold nugget' rewards buried in the ground by matching metal-detector sounds to target sounds and discriminated them from the sounds of detection for 'non-reward' objects.

Participants will be randomly assigned to train initially on **Game A** (STAR2), **B** (Submarine) or **C** (Metal Detector). Randomisation will be performed using a minimisation protocol to ensure groups are evenly matched according to tinnitus severity (Tinnitus Handicap Questionnaire score), age, and sex. The training frequency will be a pure tone within their region of normal hearing (one octave below the frequency above which they have hearing loss), that does not correspond to their dominant tinnitus pitch. Participants will train on their allocated game for 15-30 minutes per day, 5 days per week 4 weeks. After 4 week participants will cross over to different groups and train for one week on each of the other two games (Figure 1).

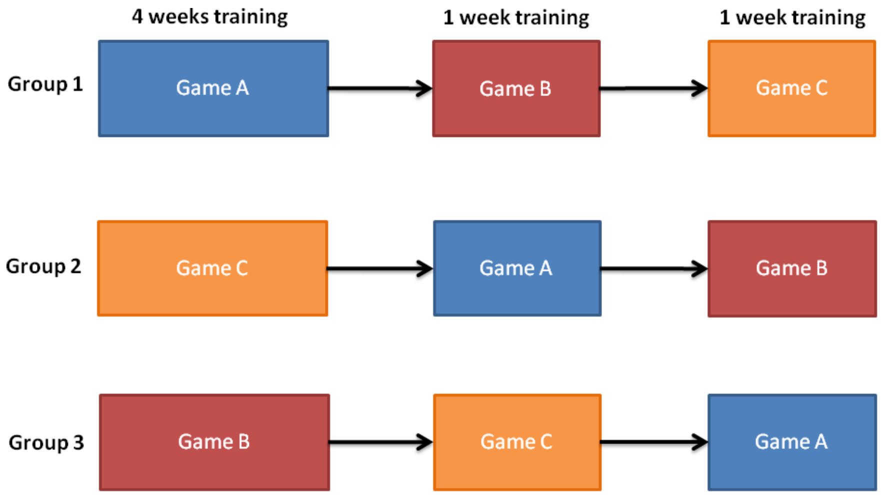

Figure 1 – Flow chart for cross-over of participants in each group.

13 Compliance

The laptops loaned for training will generate an electronic training log of how much training was done and when. Those failing to complete all the required training will provide data for useful comparisons between compliance and outcome. Furthermore, if auditory discrimination training prove effective in reducing the experience of tinnitus in this study, identifying the factors that result in limited compliance is valuable to the development of such training as an efficient clinical tool.

14 Sample Size and Justification

In our previous study it was estimated that 14 participants per group would be required to detect a difference of 420 points (assumed standard deviation of 320) in mean THQ score change between two groups. Although we found significant within-group changes, there was substantial variability in the data between groups at baseline, suggesting that a larger sample size would be more appropriate for our next study. According to Cohen (1992, 1988), to detect a large effect size difference between two independent sample means, a sample size of 26 per group would be required for a two-sided test at alpha 0.05 and a power of 0.8. For significance in a one-sided test at alpha 0.05, 20 participants per group would be required. A power calculation was performed using Tinnitus Handicap Questionnaire (THQ) score from Henry et al. (2006). When we assumed the mean difference in THQ score of 278-9 and the standard deviation of 493-5 for pre- and post- treatment differences within-subjects from our previous study group, a sample of 27 participants was required to achieve the power of 0.805 for a two-sided test at alpha 0.05. We therefore aim to recruit at least 20 and up to 27 participants per group. The upper limit would include all dropouts, which in our present study was just 7%. Recruitment will be ongoing within the project dates until these numbers are reached.

- Cohen J (1988) Statistical Power Analysis for the Behavioral Sciences (second edition). Hillsdale, NJ: Lawrence Erlbaum Associates. p.55
- Cohen J (1992) A power primer. *Psychological Bulletin*, 112, 155-159
- Henry JA, Schechter MA, Zaugg TL, Greist S, Jastreboff PJ, Vernon JA, Kaelin C, Meikle MB, Lyons KS, Stewart BJ (2006) Clinical trial to compare tinnitus masking and tinnitus retraining therapy *Acta Oto-Laryngologica,* 126, 64-69

15 Methods for Statistical Analysis

Findings will be evaluated by the statistician, chief investigator, the study coordinator and co-investigator Dr van Labeke, using statistical software (e.g. SPSS, R, SAS). The first measure of data will be a summary using descriptive statistics, i.e., analysis of the baseline characteristics of participants. The second measure of data will be a summary of within and between group comparisons with respect to the primary and secondary outcome measures using Paired t test/McNemar's test, t test, ANOVA/Kruskal–Wallis test analyses. In addition, (the simple and/or multiple) Linear regression/the General Linear Model/the Generalized Linear Model approaches will be performed, adjusting for the factors, i.e. initial severity of tinnitus, age, and gender, as appropriate for the benefit of auditory training.

16 Procedures for missing data

Patterns of missing data will be explored and a multiple imputation method will be considered to use for missing values using the MI procedure (and MIANALYZE procedure) in SAS.

17 Participant safety

None of the procedures in the study including (acoustic tests, questionnaires) are likely to cause any discomfort or adverse reaction. Participants will be required to use repetitive computer keyboard or mouse actions for short periods of time to complete the training tasks, and so will

be encouraged only to do so if the action is comfortable and they do not experience strain. If adverse effects occur at any time during the study these can be reported to any member of the research team who will proceed as is appropriate. There is also the facility for participants to be referred directly to Nottingham Audiology Services by designated members of NBRUH research staff.

18 Data Protection

The data manager is Dr Derek Hoare (Study co-ordinator). The project will abide by the Data Protection Act. The initial consent form and laptop loan form are the only documents that contain identifiable personal information and this will be stored in a locked cabinet in the office of Dr Hoare at NBRUH. Participants will be assigned a unique patient ID code that will be used on all other paper documentation. All response sheets and questionnaires that bear these ID codes will be stored in locked filing cabinets at NBRUH.

All electronic data files will be coded by the unique participant ID codes and will not contain any personal information. Files will be stored on a password protected computer system that can only be accessed by the research team. The data will be backed up on the NBRUH network on a daily basis. All data will be retained in accordance with NHS guidelines*; patient-identifying data will be kept for 10 years, non-patient identifying data will be retained for a minimum of 2 years after the study has ended to allow further analysis by the original or other research teams subject to consent, and to support monitoring by regulatory and other authorities. Data will thereafter be destroyed under confidential conditions.

*http://www.dh.gov.uk/prod_consum_dh/groups/dh_digitalassets/documents/digitalasset/dh_093027.pdf

19 Publication and Dissemination

From its earliest stages descriptive analyses of the emerging data will be presented at NBRUH science meetings. These meetings act as an informal peer-review of the research. Data from both interim and final analyses will be presented at relevant national and international conferences via oral and poster presentations.

Towards the end of the project, results will be published in high-impact peer-reviewed international journals such as the *Annals of Behavioral Medicine*. If appropriate this research will be further disseminated as magazine articles in, for example, the Journal of the British Tinnitus Association 'Quiet'. Participants will not be identified in any publications.

20 User and Public Involvement

As part of NBRUH's research strategy, research partnerships between public and researchers will be encouraged, whereby public perspective into the research process is invited and incorporated into the early stage planning and ongoing research protocols as appropriate. We have a large database of past and potential participants involved in this process, and participants from this study will be invited to partake in the design of subsequent

investigations. A number of past research participants have already volunteered their input on the usability and desirability of the games we are developing for use in this study. The new components in this study (the interactive games) will be formally piloted with more of these participants at NBRUH and/or in home-based training.

Public awareness of the study will be generated in ways to include promotion in local press and via the NBRUH website. Articles to appear in appropriate charity magazines (Deafness Research UK and the British Tinnitus Association) and representation at meetings of these charities will promote this research, and will encourage public comment and involvement in the proposed and future projects at their earliest stages.
