# Supplementary figures and images for: Gameplay as a Source of Intrinsic Motivation in a Randomized Controlled Trial of Auditory Training for Tinnitus
Source: PLoS One. 2014 Sep 12;9(9):e107430. doi: 10.1371/journal.pone.0107430 (PMC4162598; doi:10.1371/journal.pone.0107430)

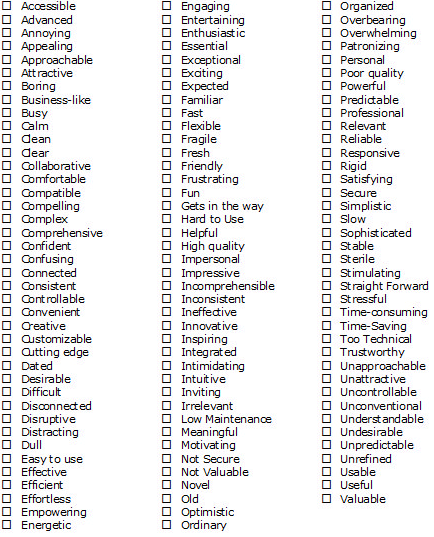

Supplement: Table S1 — Codes on product reaction cards. (TIF) [file pone.0107430.s003.tif]
